# The trickle down from environmental innovation to productive complexity

**DOI:** 10.1038/s41598-022-25940-6

**Published:** 2022-12-22

**Authors:** Francesco de Cunzo, Alberto Petri, Andrea Zaccaria, Angelica Sbardella

**Affiliations:** 1 Department of Economics and Statistics, University of Siena, Siena, Italy; 2 Institute for Complex Systems, UOS Sapienza, Rome, Italy; 3 Enrico Fermi Research Center, Rome, Italy; 4 School of Finance and Management, SOAS University of London, London, UK

**Keywords:** Physics, Statistical physics, thermodynamics and nonlinear dynamics

## Abstract

We study the empirical relationship between green technologies and industrial production at very fine-grained levels by employing Economic Complexity techniques. Firstly, we use patent data on green technology domains as a proxy for competitive green innovation and data on exported products as a proxy for competitive industrial production. Secondly, with the aim of observing how green technological development trickles down into industrial production, we build a bipartite directed network linking single green technologies at time $t_1$ to single products at time $t_2 \ge t_1$ on the basis of their time-lagged co-occurrences in the technological and industrial specialization profiles of countries. Thirdly, we filter the links in the network by employing a maximum entropy null-model. Our results emphasize a strong connection between green technologies and the export of products related to the processing of raw materials, notably crucial for the development of climate change mitigation and adaptation technologies. Furthermore, by looking at the evolution of the network over time, we observe a growing presence of more complex green technologies and high-tech products among the significant links, suggesting an increase in their importance in the network.

## Introduction

The fight against climate change is at an unprecedented critical phase: the impact of human systems of production and consumption on the environment as well as the transition to a more sustainable economy are at the center of public attention and EU policy agenda^1–3^. In this context, the development of green technologies, which despite being relatively at an early stage of the life cycle has shown a great acceleration over recent years^4^, might play a crucial role both towards containing and preventing greenhouse gas (GHG) emissions and in sustaining a shift towards less environmentally costly manufacturing processes^4–6^. It is therefore of the greatest importance to investigate how green technologies are connected to the economy and, in particular, to industrial production. This is what motivates our paper. In particular, by adopting a complexity perspective, we aim at filling some gaps in the study of the interplay between green innovation and production by implementing a highly granular analysis that allows us to explore how individual green technologies unfold into industrial production.

Several aspects of the nexus between export and green technological development have been examined at the aggregate level. By exploring different directions of causality at the firm, industry and country level, a wide array of studies has focused on the export-green innovation nexus generally highlighting a positive relationship between (policy/regulation induced) eco-innovations and export competitiveness/performance^7,8^, quality^9^, propensity^10^, or diversification^11^ (for a review on the topic with a special focus on agrifood supply chains see Galera-Quiles et al.^12^). However, previous research has largely looked at the link between overall green technological innovation and overall or sector specific export at highly aggregated levels—i.e., by focusing respectively on green patent counts and export volumes (or intensity/participation rates etc.)—overlooking the fact that a green technology may foster the export of a specific product or bundle of products, but this may not be true for all products, and a negative association with other exported goods could also be found.

Accordingly, we propose a novel quantitative framework rooted in the Economic Complexity (EC) literature^13–15^ that enables us to unpack the green innovation-export nexus by exploring how single green technological innovations, as proxied by patenting activity in climate change adaptation and mitigation technologies (CCMTs), trickle down into industrial production at the level of single products, as proxied by export data^16^. Our approach is particularly relevant when looking at green technologies, because, as they encompass different domains of know-how^17^, are designed to fulfill a broad range of functions^18^, are heterogeneous across geographical areas^19,20^ and linked in non-trivial ways to pre-existing knowledge bases^20–22^, treating them as a homogeneous aggregated corpus may fail to disentangle the possibly differentiated effects of specific green innovations on specific products. This line of reasoning is resonant with the ambition of the Economic Complexity literature to “describe and compare economies in a manner that eschews aggregation”^23^. In fact, by combining insights from the evolutionary^24,25^ and structuralist approaches^26,27^ in economics, EC describes the economy as a dynamic process of globally interconnected ecosystems and, in a departure from standard economic views, goes beyond aggregate indicators and measures of productive inputs. It considers instead a more granular view of the productive possibilities of an economy by emphasizing the importance of the composition of export baskets for long-run growth^13,28–30^. In particular, the methodology we propose is based on the Economic Fitness and Complexity (EFC) approach^15,31,32^. EFC is part of the burgeoning literature on EC and is a multidisciplinary approach to economic big data where the informational content of different types of empirical networks is maximized by using *ad hoc* algorithms which optimize the signal-to-noise ratio. EFC has proved highly successful in forecasting^30^ and explaining^33^ economic growth, and has been adopted by both the World Bank^34^ and the European Commission^35^.

Recently, some promising attempts to draw insights from the EC literature to analyse environmental issues have been put forth, with focus on environmental products^36–39^, technologies^18–20,40–43^ and jobs^44^, setting the basis for a study of the productive or technological capabilities that are relevant to the green economy. Bearing in mind the benefits and the shortcomings of using patent data for studying technological innovation and especially  their limited coverage in developing economies^45–47^, our empirical contribution builds on the Green Technology Fitness measure and green technology space proposed by Sbardella et al.^19,43^, Napolitano et al.^40^ and Barbieri et al.^20^. Moreover, our analysis is linked to studies on the coherence in firm-level patenting^48–50^, the product space^32,51^, and especially to the technology-science-export multi-partite network of Pugliese et al.^52^. However, with respect to the extant literature, the present work examines the not yet explored link between green patenting and industrial production and proposes a reliable methodology to assess the empirical connections between these two dimensions by employing a more solid network link statistical validation strategy.

In practice, the application of the EC toolbox that we propose allows us to construct a network linking single CCMTs, identified through the Y02 Cooperative Patent Classification (CPC) technology class (see “Methods” section), to single exported products, classified according to the Harmonized System (HS). This network is obtained by contracting over the geographical dimension the two bipartite networks connecting countries with comparative advantages in green technologies at time $t_1$ and countries with comparative advantages in exported products at time $t_2 \ge t_1$ respectively, with a time lag between these two layers of $\Delta T \equiv t_2-t_1$ (where $\Delta T$ could also be equal to zero). Once the co-occurrences in the same country of competitive patenting and export are identified, their statistical significance is assessed via an *ad hoc* maximum entropy null-model^53^. The final result is a green technology-product bipartite network, where each link represents the (statistically significant) conditional probability that if a generic country is proficient in a green technology $\tau$ at time $t_1$, it will also be able to export competitively product $\pi$ at time $t_2$. Each link from a green technology to an exported product highlights the fact that they share similar underlying technological and productive capabilities, therefore indicating the existence of high probability of jumping from the green technology to the linked product. An important feature of the network is its time-dependency: the direction and magnitude of the information flow can change in time and different time lags ($\Delta T$) between green patenting and product exports can be considered. Our findings show that green technologies are especially connected to the export of raw materials, such as mineral, metal, and chemical products. Their persistent presence and importance in our network resonate with the literature on the raw material requirements that the green transition entails^54–58^. In fact, materials like lithium, cobalt, indium, nickel are key inputs for several green technologies, particularly in the domain of renewable energy generation/storage and electrical mobility. Hence, to deal with the climate and environmental crisis, it is crucial to carefully take into consideration the extent to which an increase in the development of green technologies could affect mineral demand, extraction processes and environmental inequality^1,59,60^. Among the goods significantly related to green technologies we also find different products related to the export of animals and vegetables—mainly linked to technologies for GHG capture and storage—and machinery and electrical products—mainly linked to CCMTs in information and communication technologies. Moreover, a key result of our analysis is that the network structure changes when switching from $\Delta T =0$ to $\Delta T =10$, as for Δ = 10 we register a growing presence of complex green technologies and products in the statistically validated network links, suggesting that more complex green know-how requires longer to unfold into industrial production.

By shedding light on the dynamic complementarity and interrelation between green technological development and specific production lines, our methodology identifies in a quantitative and replicable way the green footprint of each product. This might prove to be instrumental in informing policy on the potential entry points in which countries can compete in emerging green markets and on the eco-innovative domains that trickle down the most into industrial production, and accordingly in designing targeted policy interventions aimed at fostering more sustainable production practices.

## Results

As mentioned above, the aim of this paper is to leverage statistically validated networks to explore the connections between green technologies and exported products, i.e. the trickle down from green technology innovation to industrial production. Each link between a green technology and a product suggests not only that being competitive in the two requires similar underlying capabilities, but also that a comparative advantage in the green technology is a good predictor for the development and succesfull export of the product. We compute the validated links for two different aggregations of the data on exported products, moving from a broader level of description—consisting of 97 so-called product chapters, labeled with 2-digit codes—to a more detailed one—consisting of 5053 product subheadings, labeled with 6-digit codes. Moreover, we are able to assess the evolution of the green technology-product network by taking into account the effect of a time lag of 10 years between the development of green technologies and the export of the products.

### Green technology—product connections

In order to build the bipartite network in which green technologies are linked to exported products, we start by considering two binary networks: the first connects countries to the green technologies they patent competitively, the second connects countries to the products they export competitively. By summing over the geographical dimension we then build the so-called *Assist Matrix*^32,52^, i.e. in our case the adjacency matrix of the green technology-exported product network, in the following way:

1 $$\begin{aligned} A_{\tau ,\pi }(t_{1},t_{2})= \dfrac{1}{u_\tau (t_{1})} \sum _c\dfrac{M_{c\tau }(t_{1}) M_{c\pi }(t_{2})}{d_c(t_{2})}, \text { with } {\left\{ \begin{array}{ll} d_{c}(t_{2}) = \sum _{\pi '}M_{c\pi '}(t_{2})\\ \\ u_{\tau }(t_{1})= \sum _{c'}M_{c'\tau }(t_{1}) \end{array}\right. } \end{aligned}$$

where the ${\textbf {M}}$ matrices define the bipartite networks where countries are linked to the green technologies or exported products in which they have a comparative advantage (see “Methods” section). That is, we are counting suitably normalized co-occurrences, with the normalization factors being the product diversification of country *c* at year $t_{2}$ $d_{c}(t_{2})$—i.e. the number of products included in the export basket of that specific country—and the ubiquity of the green technology $\tau$ at year $t_{1}$ $u_{\tau }(t_{1})$—i.e. the number of countries that are patenting in that specific technological sector. The resulting green technology-product links are then statistically validated by using the Bipartite Configuration Model^53,61^. We set at 95% the minimum significance threshold with which we validate our results, as we consider this to be a reasonable compromise between the number of observed links and their robustness. The details of the validation procedure can be found in the “Methods” section.

#### Aggregated analysis

**Figure 1:**
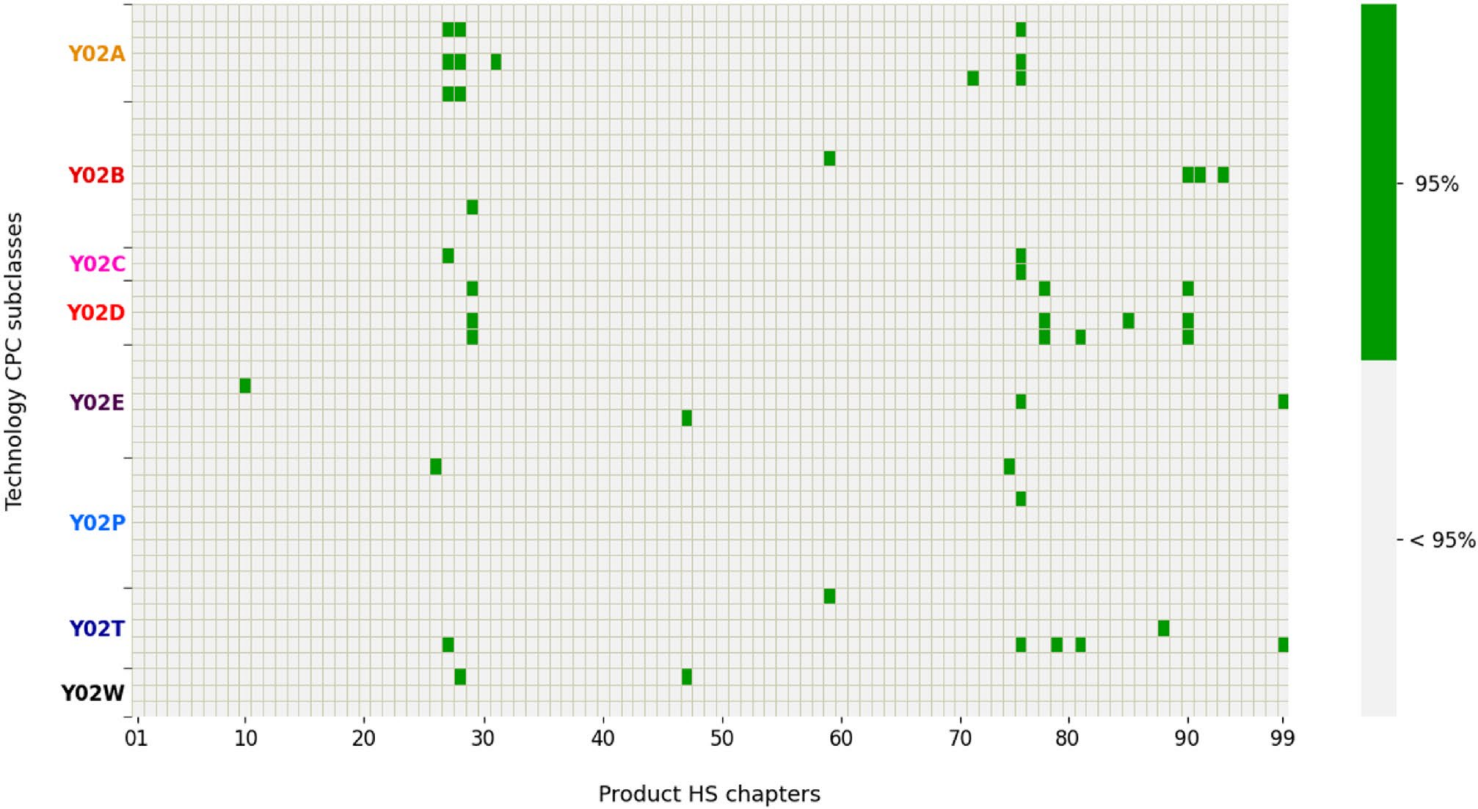
Heatmap representation of network links at 95% level of significance. Y-axis = CPC codes of green technology sub-classes; x-axis = 2-digit exported products. Each green rectangle corresponds to a link between the corresponding green technology on the y-axis and exported product on the x-axis.

Initially here we consider simultaneous normalized co-occurrences, that is with a time lag $\Delta T \equiv t_{2} - t_{1} = 0$ between the two network layers. Firstly, we investigate the links between green technologies and exported products at a 2-digit aggregation level. Figure 1 represents the adjacency matrix of the green technology-product network at a 95% statistical significance, where we find 46 significant links in total (i.e. 46 green rectangles in the figure). This figure allows us to provide some initial qualitative insights on which green technologies and exported products are connected and which are not. As regards green technologies we note that, although not uniformly, all technology sub-classes (see Table 1 for CPC Y02 code descriptions) have some links to products and are present in the network. The same cannot be said for the exported product layer: some 2-digit product sections are almost completely disconnected, including e.g. *Foodstuffs, Plastics/Rubbers, Leather* and *Textiles*, while others have a considerable amount of links. In particular, product like *Mineral fuels, Nickel, Lead, Organic* and *Inorganic chemicals* are highly connected with green technologies such as *Technologies for adaptation to climate change* (Y02A) and *CCMTs in information and communication technologies* (Y02D), indicating that a relatively high number of countries are active in both. This hints at an overlapping of the green technological know-how and the productive capabilities needed for being proficient in both, suggesting that countries that do patent in technology sub-classes as Y02A and Y02D not only are more likely to export raw material products, but also that different types of metals and chemicals are highly connected to R&D in CCMTs, and thus new sustainable avenues in their production could be explored. The topic of raw material products and a specific case study will be discussed more in detail below.

**Figure 2:**
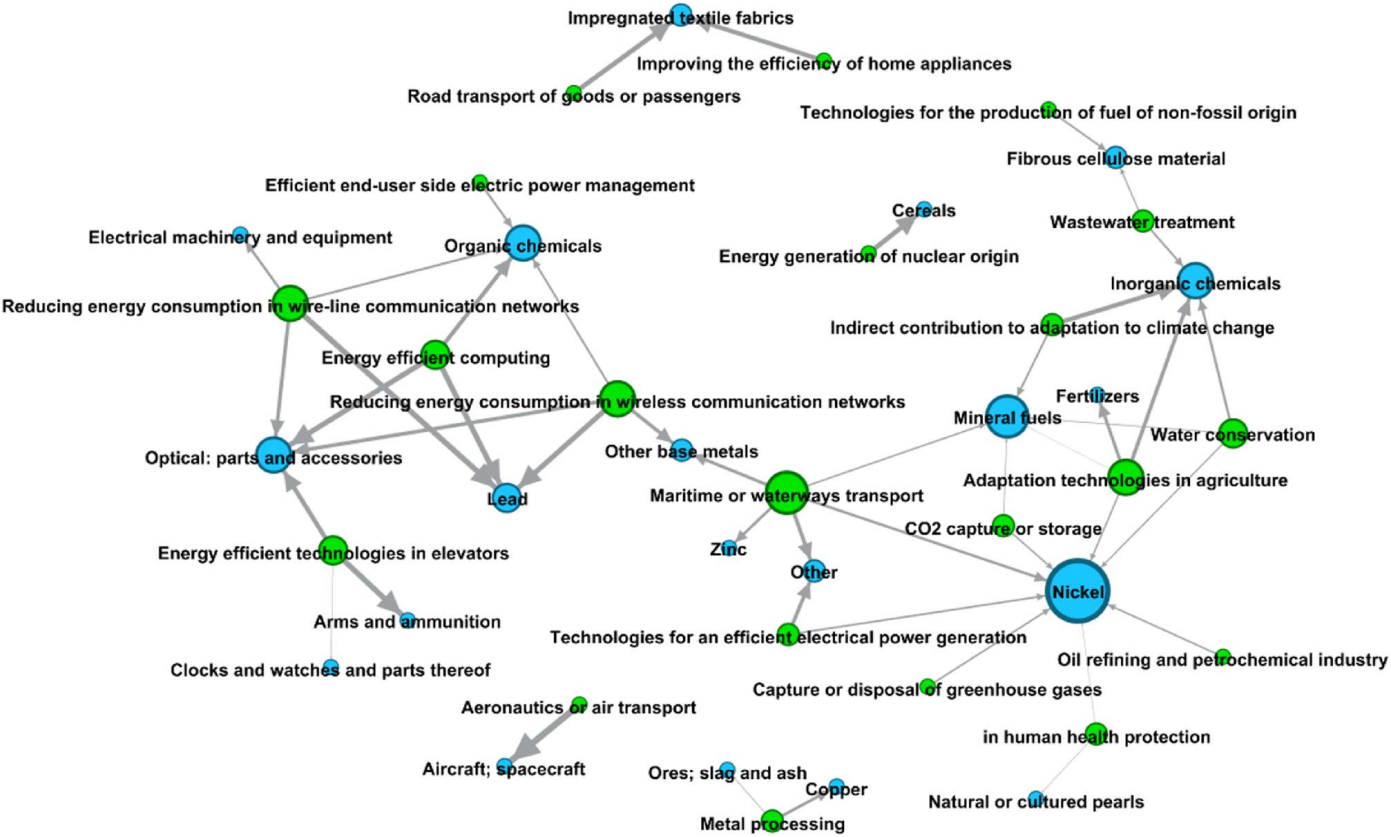
Directed network from green technologies to exported products for time lag $\Delta T = 0$ and 2-digit product aggregation level. Nodes’ size depends on their degree; edges are weighted according to the value of the Assist matrix $A_{\tau \pi }$.

In Fig. 2 we offer an alternative representation in which we show the directed network between green technologies and exported products, with the node size being proportional to the node degree and the thickness of the edges to the corresponding Assist Matrix entry. The network representation permits a clear distinction between the disconnected components (such as the two nodes relative to air transport in the bottom left) and the large connected component in the center. For instance, it is interesting to notice the energy-related cluster on the left portion of the plot, where green technologies aimed at improving efficiency in computing, in wire-line and wireless communication networks and in the electric power management are linked to the export of raw material products and optical and electrical products, which are important inputs for these kinds of technologies.

**Table 1:**
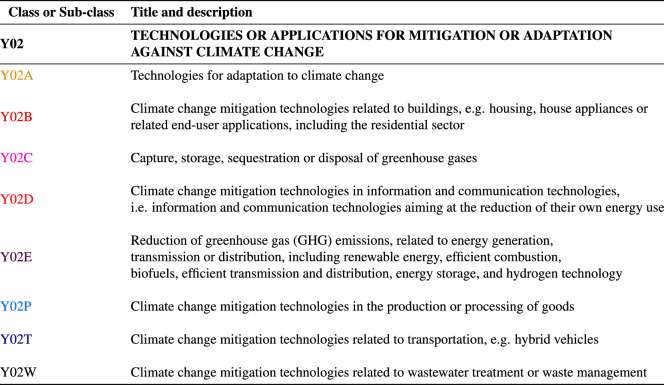
CPC Y02 tagging scheme. Source: EPO^62^.

In the first column the CPC code identifying the Y02 technology sub-class is reported. The second column reports the corresponding description.

#### Fine-grained connections

**Figure 3:**
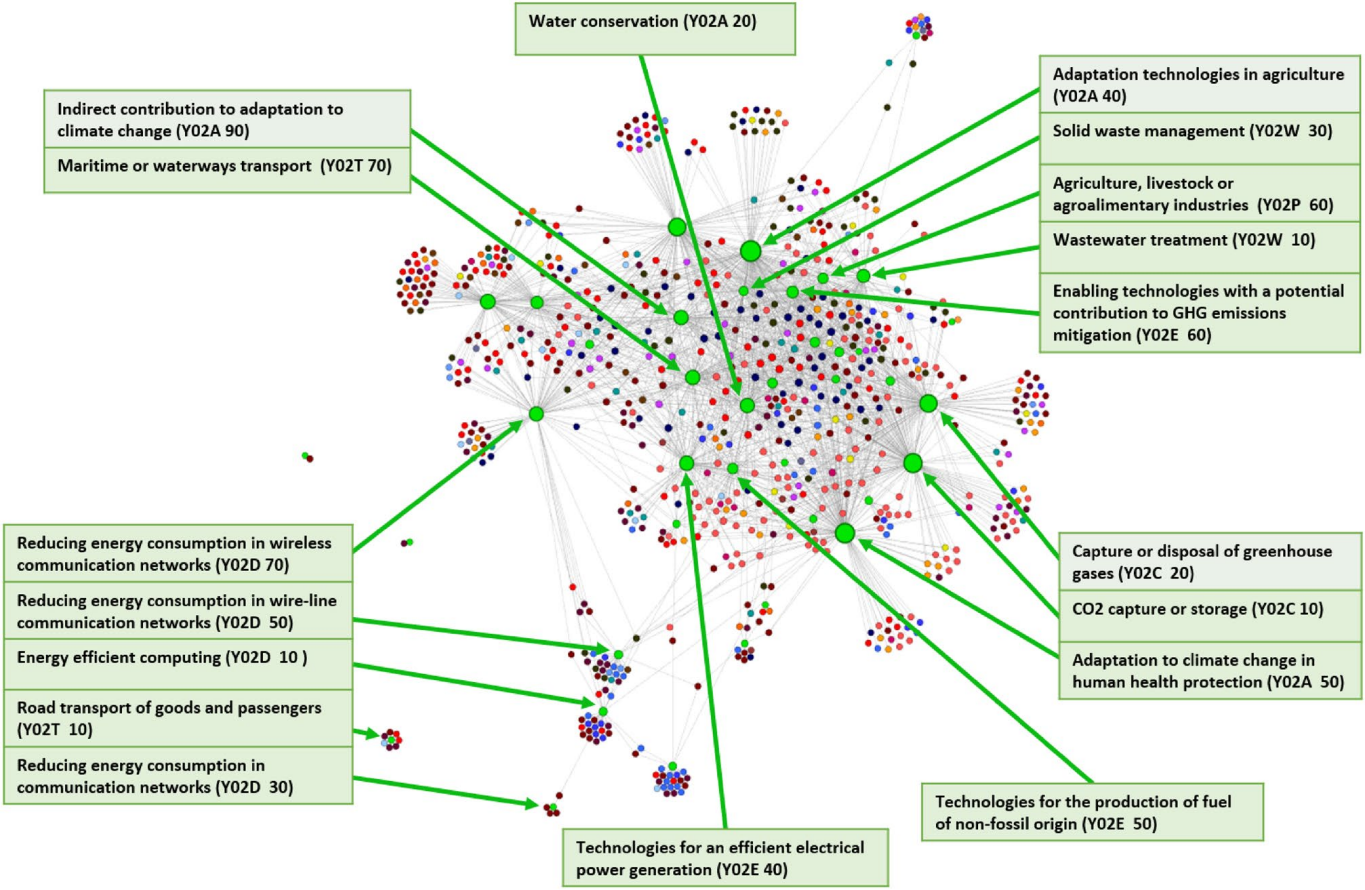
Directed network from green technologies to exported products for a time lag $\Delta T = 0$ and 6-digit products aggregation level. Nodes’ size is proportional to their degree. Green nodes: green technologies with green arrows pointing to the description of some of them. All other nodes: exported products (coloured according to Table 2).

We move forward into the analysis by considering the 5053 exported products present in the HS classification at 6-digit aggregation level. Increasing the level of data breakdown reveals the potential of our methodology, that can be easily applied to any level of data aggregation, and when applied to fine grained information can provide very punctual insights. Figure 3 represents the entire bipartite green technology-product network. The dimension of the nodes is proportional to their degree; the green ones correspond to green technologies, while all the others correspond to exported products and are coloured according to the product sections they belong to (see Table 2). We notice that, in line with the 2-digit product case, almost all green technologies (39 out of 44) are present in the network. This means that almost all green technologies are connected to the production of at least one product. However, depending on where the nodes are placed in the network, a green technology may be more or less integrated into the production system as a whole. More specifically, we can see that the periphery of the network is dominated by technologies related to services and transport, while the core of the network contains technologies belonging to sub-classes such as Y02A, which covers technologies for the adaption to the adverse effects of climate change in human, industrial (including agriculture and livestock) and economic activities, and Y02W, which covers CCMTs related to waste management.

In Table 2 we collect some descriptive information on the distribution of product nodes and edges in the network. More in detail, products belonging to primary sectors, such as animal and vegetable goods, show a large number of connections with green technologies. In particular, we observe links between different green technologies and the export of meat, fish, milling industry products and grains. All of these are largely connected with Y02A—especially with Y02A 40-*Adaptation technologies in agriculture, forestry, livestock or agroalimentary production—*and Y02A 50-*Adaptation technologies in* *human health protection* and with Y02C-*Technologies for capture, storage, sequestration or disposal of GHG*. This is consistent with the high level of pollution and emissions that the agricultural and livestock sector is accountable for^63^. Finally, consistently with the results obtained in the 2-digit product case, the subheadings belonging to minerals, chemicals and metals product sections are confirmed to be highly connected to green technologies. We elaborate on this by focusing on the export of cobalt in the following.

**Table 2:**
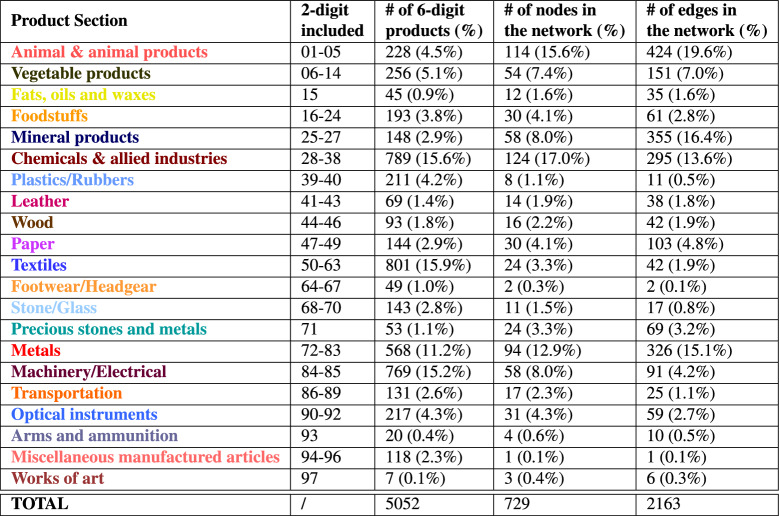
Exported product sections.

$1^{st}$ column: product section names; $2^{nd}-3^{rd}$ columns: which 2-digit products and how many 6-digit products are included. $4^{th}-5^{th}$ columns: number of nodes and edges in the network of Fig. 3. The percentages between parenthesis are computed with respect to the total values reported in the final line. Note that product *999999: Commodities not specified according to kind* is not included.

#### A case study: cobalt

**Figure 4:**
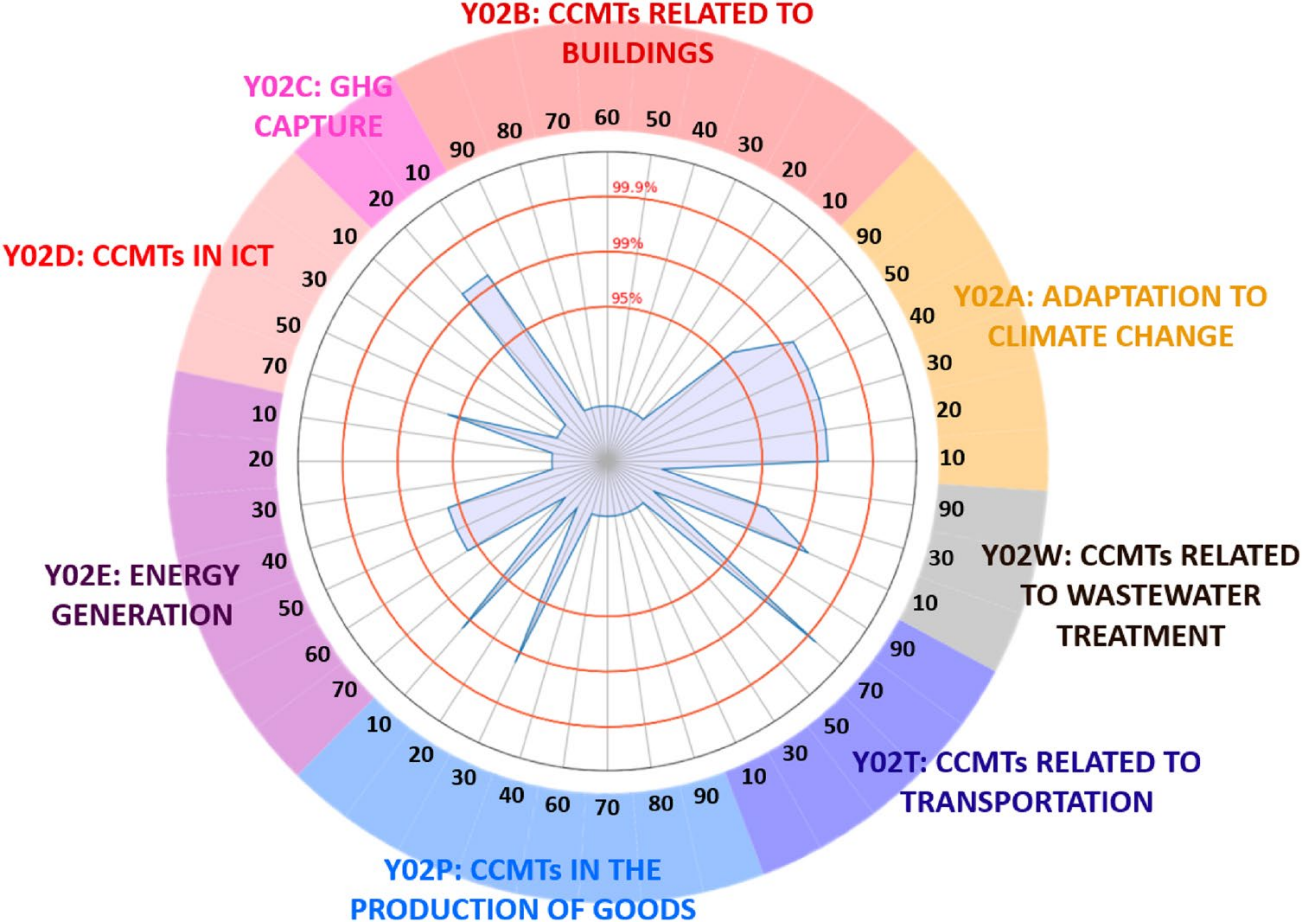
Focus on the export of *Cobalt and other intermediate products of cobalt metallurgy* (Harmonized System code 810520). Along the circular border of the figure, the CPC codes of the 44 green technology groups are labeled. Within the figure, three concentric circles delimit the significance levels of 99.9%, 99% and 95% respectively. Each peak in blue that exceeds the level delimited by one of the inner circles corresponds to a link that cobalt has with the green technology described in the border.

An interesting product export example in our green technology-product network is that of *Cobalt and other intermediate products of cobalt metallurgy *(Harmonized System code 810520). Figure 4 layout highlights which technologies are significantly connected to the successful export of cobalt, with a level of confidence even above 95%. In the figure, three red concentric circles delimit the 99.9%, 99% and 95% level of significance. The blue peaks exceeding one of these circle in the figure denote that the export of cobalt is linked at the corresponding level of significance with the green technology labeled around the circular border. In particular, cobalt export is linked with *Technologies for adaptation to climate change* (Y02A), *related to transportation* (Y02T) and *waste treatment* (Y02W), *for energy generation, transmission and distribution* (Y02E), and with *CCMTs in in information and communication technologies* (Y02D) and *in the production or processing of goods* (Y02P).

The case of cobalt is useful to stress the consistent presence of raw materials among the exported products most linked to green technologies in our network. This is far from surprising: these materials are crucial for producing green technologies, such as photovoltaic panels, wind turbines, batteries and battery energy storage systems, etcetera; indeed, an emerging literature on the topic has made different attempts to estimate the mineral intensity of green technologies and to forecast how their proliferation will shape mineral demand in the years to come^56–58,64–66^. ﻿In﻿ particular﻿, ﻿cobalt ﻿is considered a high-impact mineral for the sustainable transition and to meet expected future demand its production will need to increase up to nearly 500% of 2018 levels by 2050^55^. Cobalt is a key element in energy storage technologies, which for instance are used in the automotive sector to power electric vehicles and are needed to store energy from intermittent renewable sources, such as photovoltaic panels and wind turbines. Given that 64% of global cobalt supply comes from the Democratic Republic of Congo^67^, the risks associated with meeting its demand—which will rise if certain climate targets are to be met—and the cross-cutting way in which it is used in green technologies, have led to cobalt being placed on the European Commission’s list of critical raw materials (CRMs)^54^, which includes materials considered critical for their supply risk and economic importance. The list is updated every three years, and cobalt features in it since its first version published in 2011^68^. It is worth noticing that REGPAT, the patenting dataset we employ, does not cover the Democratic Republic of Congo. However, even if cobalt main world supplier is missing, we still observe many connections between cobalt and cobalt metallurgy products and green technologies. In particular, these connections arise from the co-occurrences of several green technologies and cobalt product exports in countries like Australia, Belgium, Canada, Finland, Norway, Russia and South Africa, which are all important producers of raw and refined cobalt^60,69^.

### Connections in a 10 year horizon

With the aim of analysing whether the spectrum of green technologies needed to gain a comparative advantage in a variety of productive sectors changes over time, here we explore how the links between green technologies and exported products change, both in qualitative and quantitative terms, moving from a time lag between the green technology and exported product layers of $\Delta T \equiv t_{2} - t_{1} = 0$ to $\Delta T = 10$. In fact, our analysis can be conducted also by considering different values of $\Delta T$ allowing for a dynamic perspective on the green technology–production nexus.

When considering $\Delta T = 10$ from a quantitative point of view we observe a slight increase in the total number of links, both in the case of 2-digit and 6-digit products (from 46 to 60 links in the case of 2-digit products and from 2166 to 2354 links in the 6-digit case). This finding is coherent with the results presented in Pugliese et al.^52^, in which the authors show that technological advancements on average anticipate export. The increase of roughly 10% of the resulting links suggests that green technologies are better integrated into the production process after a ten years digestion.

**Figure 5:**
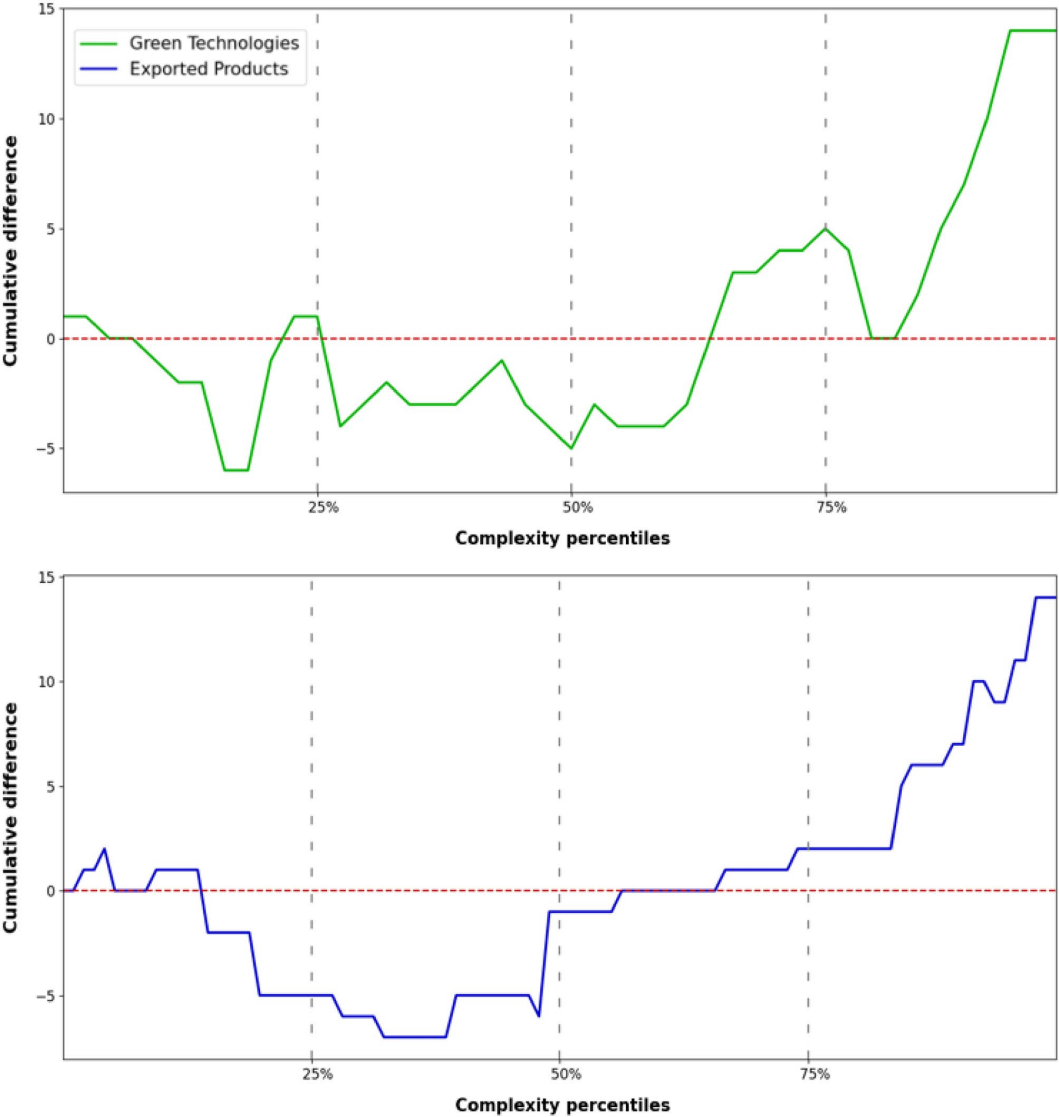
Cumulative difference between the number of node links for the time lag $\Delta T = 10$ and $\Delta T = 0$. The top panel refers to green technologies (green line), while the bottom panel (blue line) refers to 2-digit exported products. In their respective panel, green technologies and exported products are sorted in order of increasing complexity ranking. The x-axis labels 25%, 50% and 75% delimit the first, second and third quartiles of the complexity ranking (moving from the last to the first position). If the y-value is below/above 0 (dashed red line), then the cumulative number of links delimited by the corresponding green technology or product in the x-axis is higher for $\Delta T = 0/\Delta T = 10$.

Regarding possible differences in the properties of the linked technologies and products for both time lags, in Fig. 5 we plot the cumulative increment in the number of links for both green technologies and exported products. In particular, in the x-axis of the two plots we rank green technologies (top panel) and exported products (bottom panel) by increasing complexity, which is computed through the implementation of the Economic Fitness & Complexity (EFC) algorithm^15^ (see the Supplementary Information [Economic Fitness & Complexity algorithm]). The green/blue line in the figures plots the cumulative difference between the number of links that each activity shows for $\Delta T = 10$ and $\Delta T = 0$—in formula: $y_{i} = \sum_{j=last \, ranked}^{i^{th} ranked} n_{j} (\Delta T = 10) - n_{j} (\Delta T = 0)$, where $y_{i}$ is the value corresponding to the $i^{th}$ ranked green technology/product and $n_{j} (\Delta T)$ refers to the significant number of links that the $j^{th}$ ranked green technology/product has at the corresponding $\Delta T$. What emerges from the two plot layouts is significant: the new links that appear when the time lag is increased are relative to more complex products as well as to more complex green technologies. For example, we observe an increase in the number of significant links with high complexity products such as those related to the Machinery/Electrical and the Optical instruments sections and with complex climate change mitigation technologies in the following sub-classes: Y02D 10-*Energy efficient computing*, Y02D 70*-Reducing energy consumption in wireless communication networks*, Y02T 30*-Transportation of goods or passengers via railways* and Y02T 50*-Aeronautics or air transport*. Therefore, it is likely that more complex potential spillover effects in industrial production deriving from the development of a green technology will manifest themselves at a later stage over time. This is in line with the idea that more complex green technological know-how requires more time to be transmitted to the productive sectors. Moreover, this finding is in agreement with Barbieri et al.^17,21^ that study the relationship between green and non-green knowledge bases and argue that green technologies are generally complex and have a heterogeneous development process, involving different domains of know-how.

## Discussion

To address the climate crisis it will be essential to change the way economies have grown and developed^59^. Within this context, the development of eco-innovations aimed at reducing GHG emissions and their diffusion within global value chains can make important contributions towards decarbonization. However, it is important not to disregard the intrinsic limits of a “big technological fix”^70,71^ and to be aware that science and technology can indeed provide effective tools to tackle the climate change, but they will be the more effective the more they will be accompanied by a project of radical transformation of current production and development models^1,72^.

Our work might provide valuable insights on understanding possible future scenarios resulting from the development of green technologies and on how trade may act as a channel for green technology diffusion. To this end, we propose a novel application of the Economic Complexity framework and construct a network that links green technologies to exported products—at a given statistical significance, time lag, and at any CPC and HS classification aggregation level—enabling us to investigate on a case-by-case basis how green technological know-how is transmitted, even years later, into industrial production. Our empirical analysis yields two main findings. When observing simultaneous co-occurrences between comparative advantages in green technologies and exported products, we emphasise a strong association between green technologies and the export of raw materials, especially mineral and metal products. In addition, we provide evidence on a relevant number of significant connections between products belonging to the agricultural and livestock sector, among the globally highest pollutant industries^63^, such as *Animal & Animal products*, and green technologies aimed at GHG emissions capture and storage. Whereas, when considering time-lagged co-occurrences, for $\Delta T= 10$ we register a larger presence of significant links involving more complex green technologies and products (where complexity is assessed via the Economic Fitness & Complexity algorithm applied separately to products and green patents), such as green technologies related to transportation or used in ICTs, and machinery/electrical or optical instruments products. This suggests that the process that can lead to the development of the joint capabilities required for the development of complex green technologies and the competitive production of high-tech products is not instantaneous and may require years to unfold.

By emphasizing the heterogeneous, disaggregated effects that individual CCMT patents can have on the production and trade of single goods, our multi-level analysis may bear relevance to the green transition policy context. Our findings may provide support for short- and medium-term industrial policies by allowing to target, with high level of detail, green technologies that are more likely to leave larger footprints in industrial production or mitigate the impact of polluting industries on the basis of each country’s green technological capabilities. Accounting for differentiated effects also over time through the dynamic observation of the green technology-product network, our approach might be of help in uncovering the time window required by more complex green technological know-how to be transmitted into production, and thus in designing policies acting on different time horizons. Furthermore, since monitoring the trade of environmental goods is a central objective on the global policy agenda^73,74^, by identifying green footprints in products, our work might contribute to classifying environmental products. In fact, whilst the introduction in the Harmonized System of several 6-digit subheadings including new environmental goods was announced^75^ in 2020, the updated classification is not yet available, and currently a clear-cut identification of environmental goods within existing product classifications constitutes a difficult task—as for instance it is impossible to distinguish between combustion engine and electrical cars.

With respect to the Economic Complexity literature that focuses on various aspects of the green economy, this work introduces different elements of novelty. In fact, previous works analyse green technologies and industrial production separately, either without exploring the connections between green patents and exported products or by analysing it ex-post^19,36,38,39,41^. Moreover, extant research^22,37–39,41^ proposes a number of versions of the green product or technology space, however without considering any dynamic element, as well as without using any validation strategy of network links and thus possibly considering spurious associations, that fail to account for the ubiquity of products/technologies and the diversification of countries, as we are instead able to do in this contribution.

This paper opens up different possibilities of extension of our empirical framework that might contribute to broadening our understanding of the complex interactions that the path towards the sustainability transition entails. First, we believe it would be of interest to explore the interplay of green technological and productive capabilities with other important dimensions of human activity, in particular by looking at the relationship between green technology development, industrial production and (1) the labour market (including e.g. data on employment and wages at sectoral and occupational levels); (2) the scientific production of countries through academic publication data^76^. Second, if new data will become available, analysing longer time spans might increase the observed signal^52^, thus helping to better characterise the structural relationships that link green technologies to production. Third, by geolocalising the co-occurrences that we have identified, we plan to define a measure of green technology-product relatedness that might shed light on the green footprints in the specialisation profiles of each country or region. Finally, as mentioned above, our findings call attention to the strong connection between the development of green technologies and the trade of metals and minerals they require to be successfully realized and deployed. The critical raw materials intensity of these technologies is a core issue in the policy debate^55,56,68^: CRM extraction contributes importantly to GHG emissions^57,77,78^, with the risk of thwarting the efforts towards the promotion of less polluting energy sources by shifting emissions upstream in the energy generation process and increasingly relocating environmental negative externalities in the Global South^60,64,79^. Accordingly, future research should delve deeper into such CRM dependency. Our next project points in this direction and aims at mapping mineral and metal inputs in green technologies through keyword search on patent texts. On a larger scale, we believe it would be of paramount importance to direct future research and policy towards preserving the stability of the raw materials value chain by limiting the supply dependence on and the over exploitation of specific areas, as well as promoting recycling practices, more transparent and fairer raw material extraction activities, while also fostering the development of eco-innovations less dependent on critical raw materials.

## Methods

### Data

We use data on patent applications in environment-related domains as a proxy for environment-related innovation, and data on exported products as a proxy for production^16^. Both datasets consist of single data collections recorded annually at a country level. We use information on patent applications on 44 green technological fields—corresponding to the Coperative Patent Classification groups listed in the Supplementary Information [Table S2:CPC detailed descriptions]—for 48 countries between 1995 and 2019; and on product exports—classified according to the Harmonized System and whose number depends on the level of aggregation considered: 97 in the 2-digit case, 5053 in the 6-digit one—measured in US dollars for 169 countries between 2007 and 2017. As explained more in detail in the next section, our methodology requires selecting the countries in common between the two data collections, which turn out to be 47. All data can be represented as matrices: we denote by ${\textbf {W}}(t)$ and ${\textbf {V}}(t)$ the matrices corresponding respectively to the data on green patents and exported products in year *t*. Each matrix has a number of rows and columns equal to the number of countries *c* and activities *a* respectively, where the latter refer to either green technologies $\tau$ and exported products $\pi$. A more comprehensive description of the two datasets we use, including also a list of all countries at our disposal, is reported in the Supplementary Information [Data features].

### Data preprocessing

#### Temporal aggregation

The information on both exported products and the patented inventions is collected yearly; it is then possible to investigate the connections at different time scales. While annual data can offer more detailed results, i.e. distinct for each year considered, it may also supply them with more noise. In fact, data can fluctuate significantly from one year to another. In order to minimize the possibility that the detected green technology-product connections are the result of data fluctuations, we consider the total volume of products and patents produced in given time intervals. For our analysis, we compute the matrices ${\textbf {W}}(\delta ,t)$ and ${\textbf {V}}(\delta ,t)$, corresponding to the time interval of $\delta$ years ending in the year *t*. To this aim, we sum the yearly matrices ${\textbf {V}}(t)$ and ${\textbf {W}}(t)$ over $\delta$:

2 $$\begin{aligned} \begin{aligned} {\textbf {V}}(\delta ,t)&= \sum _{t'=t-\delta +1}^{t} {\textbf {V}}(t')\\ {\textbf {W}}(\delta ,t)&= \sum _{t'=t-\delta +1}^{t} {\textbf {W}}(t') \end{aligned} \end{aligned}$$

Summing data over a time window of $\delta$ years reduces the noise in our results, giving more weight to patents and exports that are consistently registered several times in nearby years. ﻿Given the years present in the employed datasets, we sum the matrices over 5 years ($\delta = 5$). Starting from the layer of exported products, we select the two most recent 5-year aggregate matrices available to us, with the condition that the years included in the two sets are not overlapping. Therefore, the two resulting matrices are ${\textbf {V}}(\delta ,t)=\{{\textbf {V}}(5,2012); {\textbf {V}}(5,2017)\}$. Next, depending on which time lag $\Delta T$ we consider between the two layers, we select the green patents matrices. Thus, for the time lag $\Delta T = 0$, the corresponding matrices are ${\textbf {W}}(\delta ,t)=\{{\textbf {W}}(5,2012); {\textbf {W}}(5,2017)\}$, while for $\Delta T = 10$, when we consider green patenting as a “predecessor” of exporting, they are ${\textbf {W}}(\delta ,t)=\{{\textbf {W}}(5,2002); {\textbf {W}}(5,2007)\}$. To simplify the notation, hereinafter we omit the $\delta$ dependency of the data matrices, however all our results are produced from the analysis of the aggregated 5-year data collections mentioned above. Choosing the most recent time frame available in the data allows us to obtain more relevant implications from our work. However, to avoid any possible bias due to our choice of time window, we have conducted different robustness checks on the network links using both different aggregation time intervals $\delta$ and final year *t,* and we have concluded that the green technology-product links we find are robust to such changes in the parameters. These tests can be found in section *Robustness test* of the Supplementary Information.

#### Revealed comparative advantage

Both exports and patents’ matrices strongly depend on the total size of the economy or sector. In order to remove this size correlation, we compute Balassa’s Revealed Comparative Advantage (RCA)^80^ of both activities. The RCA is computed as the ratio between the weight of activity *a* (be it a patent in a technology field $\tau$ or the export of a product $\pi$) in the portfolio of country *c* and the weight of that same activity with respect to the world volume, as reported in the following equation:

3 $$\begin{aligned} RCA_{ca}= \dfrac{\dfrac{X_{ca}}{\sum _{a'}X_{ca}}}{\dfrac{\sum _{c'}X_{c'a}}{\sum _{c'a'}X_{c'a'}}} \end{aligned}$$

where the element $X_{ca}$ refers to both $W_{c\tau }$ and $V_{c\pi }$, i.e. the elements of the country-green technology and country-exported product matrices (for a more detailed description on how the matrices are built, we refer to the Supplementary Information [Data Features]). The next step is the computation of the binary matrices ${\textbf {M}} = {\textbf {M}}_{ca} = \{{\textbf {M}}_{c\tau }; {\textbf {M}}_{c\pi }\}$, whose elements are set to 1 if the value of $RCA_{ca} \ge 1$ and to 0 otherwise, i.e. when that country *c* is not competitive in activity *a*. The RCA metric is frequently used in the Economic Complexity framework to assess whether a country is a significant exporter of a product^14,51^. The extension of its use to the patent layer^52^ allows us to compare patent and export data in a coherent way as presented in the following sections.

### Construction of the validated network

#### Full technology-product network

Starting from the binary matrices ${\textbf {M}}$ described above, that summarise the comparative advantages in the products and technologies of different countries, a network linking green technologies to products can be derived. The method adopted here has been widely exploited in the Economic Complexity framework^52^: the idea is to count how many countries have competitively developed a given green technology at time $t_1$ and are also competitive in the export of a product at time $t_2$. This number thus quantifies the empirical green technology-product co-occurrences^81^. In practice, however, the co-occurrences should be suitably normalized to take into account the nested structure of the bipartite networks: countries with high diversification $d_c$ and technologies with high ubiquity $u_\tau$ provide less information and for this reason the weight of the corresponding co-occurrences is lowered. The result of this normalization is called Assist Matrix^32,52^. The co-occurrences can be obtained from the contraction of the binary country-technology and country-product matrices. The assist matrix element $A_{\tau \pi }$ depends on both the year $t_{1}$ relative to the patenting of the technology $\tau$ and the year$t_{2}$ of the subsequent export of product $\pi$. In formula:

4 $$\begin{aligned} A_{\tau \pi }(t_{1},t_{2})= \dfrac{1}{u_\tau (t_{1})} \sum _c\dfrac{M_{c\tau }(t_{1}) M_{c\pi }(t_{2})}{d_c(t_{2})}, \text { with } {\left\{ \begin{array}{ll} d_{c}(t_{2}) = \sum _{\pi '}M_{c\pi '}(t_{2})\\ \\ u_{\tau }(t_{1})= \sum _{c'}M_{c'\tau }(t_{1}) \end{array}\right. } \end{aligned}$$

By counting the co-occurrences between green technologies and exported products—while weighing them with the degree (or ubiquity) of the green technology $u_{\tau }$ and the country degree (or diversification) in the exports $d_{c}$—each element of the matrix $A_{\tau \pi }(t_{1},t_{2})$ provides a quantitative measure of how likely is to have a comparative advantage in exporting product $\pi$ in year $t_{2}$, conditional on having a comparative advantage in green technology $\tau$ in year $t_{1}$. Therefore, $t_{1}$ and $t_{2}$ indicate that in the formula it is considered the possibility that the link couples patents developed in a given year with products exported in a different year. Finally, it is important to notice that while a statistically significant link between a green technological class and a product is established on the basis of the empirical conditional probability that having a comparative advantage in the green technology will lead to a comparative advantage the export of a specific product, we are in no way arguing that there is a causal relationship that links green patenting to subsequent product export. After the computation of the Assist Matrix, we statistically validate the empirical results expressed by each node $A_{\tau \pi }(t_{1},t_{2})$ through the implementation of a null model which we present in the following section.

#### Statistical validation of the network using a null model

The matrix elements computed in Eq. (4) need to be validated by a statistical test able to distinguish meaningful links from noise and to supply a confidence level for assessing the probability that two nodes share a statistically significant number of co-occurrences. In particular, here we rely on the filtering procedure, based on the Bipartite Configuration Model (BiCM)^61^, developed by Saracco et al.^53^ for the projection of bipartite networks into monopartite networks, and subsequently adapted to a multi-partite setting by Pugliese et al.^52^. It must be however noted that no absolute criteria exists for the choice of the model, and that different null models can yield different outcomes^82^. Here, we use a null model for the binary matrices ${\textbf {M}}$, in which the matrices are randomised except for some constraints we impose^83^—in this case the average degrees of the nodes. The use of BiCM allows for a stricter filtering procedure with respect to other null models^82^ and correctly takes into account the possible noise present in the input data^53,82,83^. This class of models is based on the maximum entropy principle^84^, which leads to the realisation of an ensemble $\Omega$ of bipartite networks $\tilde{{\textbf {M}}}$, where links are random but maximize the number of possible configurations which satisfy the imposed constraints. In the present case the entropy function:

5 $$\begin{aligned} S = -\sum _{\tilde{M} \in \Omega } P(\tilde{M})\ln P(\tilde{M}) \end{aligned}$$

is maximized under the constraint that the ensemble averages $\langle \dots \rangle _\Omega$ of the ubiquity of activities *a* (i.e., of green technologies $\tau$ and exported products $\pi$) and of countries diversification of the random networks, $\tilde{u}_a(t)$ and $\tilde{d}_c(t)$ respectively, must be equal to the observed ones (labeled without the tilde symbol):

6 $$\begin{aligned} \begin{array}{ccc} \langle \tilde{d}_c(t) \rangle _\Omega &{} = &{} d_c(t) \\ \langle \tilde{u}_a(t) \rangle _\Omega &{} = &{}u_a(t) \end{array} \end{aligned}$$

Hence, these networks are random but preserve the information present in the empirical degrees.

The maximization procedure yields a probability distribution for each possible pair of country-activity nodes to be linked. Then, we use them to perform a direct sampling of the ensemble $\Omega$. The ensemble is composed of a number of realisations of the null model; the number of realizations is established by considering the *p*-value threshold with which we choose to validate the links in the technology-product network. In particular, since our results are mostly set to a statistical significance of 95%, we construct ensembles consisting of 10000 realisations of the null model. In such a way, a rough but conservative estimate yields a sampling error of 5 ‰. For each pair of null model realizations $\{\tilde{M}_{c\tau }(t_{1}); \tilde{M}_{c\pi }(t_{2})\}$ related to the green technology and exported product layers, we compute the corresponding null Assist Matrix of element $\tilde{A}_{\tau \pi }(t_{1},t_{2})$ through a contraction as in Eq. (4) and therefore build an ensemble of 10000 realizations of null Assist matrices. Finally, for each possible green technology-product $\tau$-$\pi$ link we compare the empirical value $A_{\tau \pi }(t_{1},t_{2})$ with the 10000 null values of that same link. We are thus able to assess the statistical significance of our results: for example, if we want to select 95% significant links, we consider only links those with the empirical value higher than the corresponding null ones in at least 9500 cases out of 10000.

#### Validation of the results for a specific time lag

As already stressed, our methodology allows us to build different networks linking green technologies to exported products by varying the temporal dimension. We express the time dependence of the analysis through the time lag $\Delta T$, the difference between the year $t_{2}$ of the country-product matrix and the year $t_{1}$ of the country-green technology matrix. Given the years present in the two data collections we employ, in our analysis we consider two time lags: $\Delta T= 0$ and $\Delta T = 10$. We recall that our matrices refer to sums over 5-year intervals. To each of the two considered time lags we associate two different pairs of 5-year aggregate technology-product matrices: ${\textbf {W}}(2012)-{\textbf {V}}(2012)$ and ${\textbf {W}}(2017)-{\textbf {V}}(2017)$ for $\Delta T = 0$; ${\textbf {W}}(2002)-{\textbf {V}}(2012)$ and ${\textbf {W}}(2007)-{\textbf {V}}(2017)$ for $\Delta T = 10$, where, by following equation (2), the number in parenthesis represents the last year in the five year interval. For each pair of matrices we follow all the steps described above—i.e., RCA and Assist Matric computation, and statistical validation of the links through the null model at a selected *p*-value—and we consider only the links that are statistical significant in both of them. For instance, the links represented in Fig. 2 are those that show 95% statistical significance in both the networks obtained from ${\textbf {W}}(2012)-{\textbf {V}}(2012)$ and ${\textbf {W}}(2017)-{\textbf {V}}(2017)$. Therefore, we consider two levels of significance to validate our results. The first is the assessment of the links’ statistical significance through the null model that allows us to assign a confidence interval within which we exclude that the links are solely the result of noise. The second is the condition according to which we only consider links validated at a certain statistical threshold in both the pairs of green technology-product matrices for the selected $\Delta T$: we believe this to be an important step for arguing that the know-how of a specific technology is transmitted to a product immediately or requires a time lag of 10 years, regardless of the specific years we are considering. Finally, it provides additional robustness to the analysis of our network beyond the adoption of the null model.

## Supplementary Information

Supplementary Information.

## Data Availability

The data that support the findings of this study are available from https://www.oecd-ilibrary.org/science-and-technology/the-oecd-regpat-database_241437144144 (REGPAT) and https://comtrade.un.org/ (UN COMTRADE), but restrictions apply to the availability of these data, which were used under license for the current study, and so are not publicly available. Processed data on coarse-grained Assist matrices are however available from the corresponding authors upon reasonable request.
