# Supplementary material for: The trickle down from environmental innovation to productive complexity
Source: Sci Rep. 2022 Dec 22;12:22141. doi: 10.1038/s41598-022-25940-6 (PMC9780348; doi:10.1038/s41598-022-25940-6)
Supplement: Supplementary file 1 — Supplementary Information. [file 41598_2022_25940_MOESM1_ESM.pdf]

The trickle down from environmental innovation to productive complexity

SUPPLEMENTARY INFORMATION

Francesco de Cunzo[1], Alberto Petri[2], Andrea Zaccaria[2,*], and Angelica Sbardella[3]

[1]University of Siena, Department of Economics and Statistics, Siena, Italy
[2]Institute for Complex Systems, UOS Sapienza, Rome, Italy
[3]Enrico Fermi Research Center, Rome, Italy

[*]andrea.zaccaria@cnr.it

1 Data Features

1.1 Green Patents

In this paper we look at patent data as a proxy of environment-related innovation (to which we will also refer to as green technologies) that is increasingly becoming the golden standard in the literature to measure green innovative activities, as it is widely available, it can provide an array of quantitative information on the nature of the invention and its applicant or inventor, including their geographical location, affording in such away to easily geo-localise patents both at country and local levels [1]. Moreover, and very importantly, patent data can be disaggregated into increasingly fine-grained technological areas, allowing very specific green technologies to be identified, also through keyword searches [2]. Green technology is particularly interesting because it shows distinctive features with respect to non-green technologies, appears to be heterogeneous and encompasses many domains of know-how. It has in fact been proven that the knowledge generation process behind the development of these technologies substantially differ from non-green ones [3] and across geographical areas [4], but is linked in non-trivial ways to the pre-existing knowledge base [5].
As a response to the increasing attention and concern about climate change and renewable energy generation, we are witnessing a large increase of patent applications in environment-related domains: according to the European Patent Office (EPO), in the last years there have been around 1.5 million patent applications in sustainable technologies [6]. Searching for environment-related patent documents has, therefore, been a challenge, especially because in the past documents relating to sustainable technologies did not fall into one single classification. In 2013 the EPO and the United States Patent and Trademark Office (USPTO) agreed to harmonise their patent classification practices and developed the Cooperative Patent Classification (CPC) system, which encompasses five hierarchical levels spanning from 9 sections to around 250000 subgroups and where codes starting with the letters A to H represent a traditional classification of innovative activity in technological fields, while the Y section [7] tags cross-sectional technologies. Here in particular we employ the Y02–*Technologies or applications for mitigation or adaptation against climate change* retrieved from the OECD REGPAT database [8]. The Y02 class consists of more than 1000 tags organised in 9 sub-classes and includes patents related to climate change adaptation and mitigation (CCMT)[1] technologies concerning a wide range of technologies related to sustainability objectives, such as energy efficiency in buildings, energy generation from renewable sources, sustainable mobility, smart grids and many others, the details of which can be found in Table 2 below and, in a more synthetic fashion, in Table 1 of the manuscript.
Following the notation given in the manuscript, we have matrices $\mathbf{W}(t)$ from 1995 to 2019. The number of countries (i.e. the number of rows in each matrix) are 48 (see Table 1). The number of columns are 44 technological fields corresponding to the CPC groups listed in Table S2. To build such matrices, each patent family — i.e. each collection of patent applications covering the same or similar technical content — counting

[1]According to the United Nations Environmental Program (UNEP): "Climate Change Mitigation refers to efforts to reduce or prevent emission of greenhouse gases. Mitigation can mean using new technologies and renewable energies, making older equipment more energy efficient, or changing management practices or consumer behavior"[9]. However, it is important to notice that mitigation does not necessarily goes hand in hand with sustainable and "green" practices. Some CCMTs, such as nuclear technologies, might also pose threats on the environment or be polluting.

as a unit and recorded in REGPAT is divided between all technology codes τ and all countries c with which it is associated, following the procedure adopted in Napolitano et al. [10] and Barbieri et al. [11]. Therefore, each element $W_{c\tau}(t)$ of the matrix represents the fraction of patent families associated with the country-technology pair $c - \tau$ in year t.

1.2 Exported products

For the export data we resort to the UN-COMTRADE database [12], which provides the yearly trade flows between countries, expressed in US Dollars. This information is provided at the product level, so that it is possible to study in detail which countries are exporting a given amount of a given product in a chosen year. The products in the dataset are classified according to the Harmonized System, a hierarchical classification that allows to disaggregate the economic sectors from two digits (about 100 different product chapters) up to six digits (about 5000 different product subheadings) codes. This degree of freedom is key to investigate the effect of technological innovations at different levels of detail: in fact, we move from the links that green technologies have with the export of entire product categories such as those related to the Machinery/Electrical sector to those that they have with the export of detailed single products such as electric motors. We point out that since importers' and exporters' declarations do not precisely coincide, suitable reconstruction algorithms are needed in order to achieve a coherent and cleaned dataset. In order to do so, we adopt a global Bayesian optimization approach to obtain a denoised dataset, as proposed by Mazzilli et al. [13]. The goodness of this procedure is empirically confirmed by Tacchella et al. [14], who, by employing the denoised dataset, obtained a sizeable increase in GDP forecasting performance.

From the trade flows we obtain the export matrices $\mathbf{V}(t)$, where t ranges from 2007 to 2017: the number of rows, corresponding to the number of countries, is equal to 169 (see Table S1), while the number of columns, corresponding to the exported products, depends on the level of aggregation considered (97 in the 2-digit case, 5053 in the 6-digit one). Thus, each element $V_{c\pi}(t)$ represents the volume of exports of the product π, expressed in thousands of dollars, by the country c in year t.

1.3 Country list

Depending on which step of our analysis we deal with, we consider all countries included in each collection or only those in common. In particular, the computation of the Revealed Comparative Advantage (RCA) is done separately for patents and exports, thus including all countries in the respective datasets. On the contrary, the calculation of the assist matrix is done by contracting the patent and export data over the geographical dimension, and therefore we only consider those in common. In Table S1 we collect all the countries included in both datasets, also writing their names in different colours depending on whether they are part of the 47 common countries between the two datasets or they are only present in one of them.

Country full list			
Afghanistan	Albania	Algeria	Andorra
Angola	Argentina	Armenia	Australia
Austria	Azerbaijan	Bahrain	Bangladesh
Belarus	Belgium	Belize	Benin
Bhutan	Bolivia	Bosnia Herzegovina	Botswana
Brazil	Brunei	Bulgaria	Burkina Faso
Burundi	Cambodia	Cameroon	Canada
Cape Verde	Central African Republic	Chad	Chile
China	Colombia	Congo	Costa Rica
Croatia	Cuba	Cyprus	Czech Republic
Democratic Republic Congo	Denmark	Dominican Republic	Ecuador
Egypt	El Salvador	Equatorial Guinea	Eritrea
Estonia	Ethiopia	Fiji	Finland
France	French Polynesia	Gabon	Gambia
Georgia	Germany	Ghana	Greece
Greenland	Guatemala	Guinea	Guinea-Bissau
Guyana	Haiti	Honduras	Hungary
Iceland	India	Indonesia	Iran
Iraq	Ireland	Israel	Italy
Ivory Coast	Jamaica	Japan	Jordan
Kazakhstan	Kenya	Kuwait	Kyrgyzstan
Laos	Latvia	Lebanon	Lesotho
Liberia	Libya	Liechtenstein	Lithuania
Luxembourg	Macedonia	Madagascar	Malawi
Malaysia	Maldives	Mali	Malta
Mauritania	Mauritius	Mexico	Moldova
Mongolia	Montenegro	Morocco	Mozambique
Myanmar	Namibia	Nepal	Netherlands
New Zealand	Nicaragua	Niger	Nigeria
North Korea	Norway	Oman	Pakistan
Panama	Papua New Guinea	Paraguay	Peru
Philippines	Poland	Portugal	Qatar
Romania	Russia	Rwanda	Saudi Arabia
Senegal	Serbia	Seychelles	Sierra Leone
Singapore	Slovakia	Slovenia	Somalia
South Africa	South Korea	South Sudan	Spain
Sri Lanka	Sudan	Suriname	Swaziland
Sweden	Switzerland	Syria	Tajikistan
Tanzania	Thailand	Togo	Tunisia
Turkey	Turkmenistan	Uganda	Ukraine
United Arab Emirates	United Kingdom	Uruguay	USA
Uzbekistan	Venezuela	Vietnam	Yemen
Zambia	Zimbabwe		

Table S1: All country list.
Legend: "Red-labelled country": included in both datasets (47 in total); "Green-labelled country": included in green patents dataset only (1 in total); "Black-labelled country": included in exported products dataset only (122 in total).

2 Table S2: Y02-CPC detailed descriptions

As mentioned, we employ the Y02 class of the CPC patent classification to identify climate change mitigation technologies and we thus have information on patent applications for 44 green technology groups. These are in turn grouped into 8 subclasses, which are reported in Table 1 of the manuscript. In Table S2, we report the codes and descriptions at the group aggregation level.

CPC subclass		Description
Y02A	10	Adaptation to climate change at coastal zones
	20	Water conservation
	30	Adapting infrastructure
	40	Adaptation technologies in agriculture
	50	in human health protection
	90	Indirect contribution to adaptation to climate change
Y02B	10	Integration of renewable energy sources in buildings
	20	Energy efficient lighting technologies
	30	Energy efficient heating
	40	Improving the efficiency of home appliances
	50	Energy efficient technologies in elevators
	60	ICT aiming at the reduction of own energy use
	70	Efficient end-user side electric power management
	80	Improving the thermal performance of buildings
	90	GHG emissions mitigation [Buildings]
Y02C	10	CO2 capture or storage
	20	Capture or disposal of greenhouse gases
Y02D	10	Energy efficient computing
	30	Reducing energy consumption in communication networks
	50	Reducing energy consumption in wire-line communication networks
	70	Reducing energy consumption in wireless communication networks
Y02E	10	Energy generation through renewable energy sources
	20	Combustion technologies with mitigation potential
	30	Energy generation of nuclear origin
	40	Technologies for an efficient electrical power generation
	50	Technologies for the production of fuel of non-fossil origin
	60	Enabling technologies
	70	Other energy conversion systems reducing GHG emissions
Y02P	10	Metal processing
	20	Chemical industry
	30	Oil refining and petrochemical industry
	40	Processing of minerals
	60	Agriculture
	70	CCMT in the production process for final products
	80	CCMT for sector-wide applications
	90	GHG emissions mitigation [Production]
Y02T	10	Road transport of goods or passengers
	30	Transportation of goods or passengers via railways
	50	Aeronautics or air transport
	70	Maritime or waterways transport
	90	GHG emissions mitigation [Transportation]
Y02W	10	Wastewater treatment
	30	Solid waste management
	90	GHG emissions mitigation [Wastewater]

Table S2: Descriptions of environmental technology groups. In the first column (divided in turn into two sub-columns) the CPC code identifying the technology group is reported. The second column adds the corresponding group descriptions.

3 Economic Fitness & Complexity algorithm

In Fig. 5 of the mauscript we order the codes related to green technologies and exported products according to their level of complexity. The latter is intended as an algorithmic assessment of the number and the sophistication of the capabilities needed to be competitive in a given activity. To compute it, we use the Economic Fitness & Complexity (EFC) algorithm product [15, 16], originally introduced for exports but also applied to green patents [4]. More in detail, it consists of a non-linear iterative algorithm that, starting from the binary matrices $\mathbf{M}_{ca}(t)$ obtained through the implementation of RCA detailed in the manuscript in the Methods section, allows to quantify the complexity of the activities Q_a and the competitiveness of the countries, namely their fitness F_c, that perform in them. The mathematical formulation of the algorithm at each iteration n is as follows:

$$\begin{cases} \tilde{F}_c^{(n)} & = \sum_a M_{ca} Q_a^{(n-1)} \\ \tilde{Q}_a^{(n)} & = \dfrac{1}{\sum_c M_{ca} \dfrac{1}{F_c^{(n-1)}}} \end{cases} \rightarrow \begin{cases} F_c^{(n)} & = \dfrac{\tilde{F}_c^{(n)}}{\left\langle \tilde{F}_c^{(n)} \right\rangle_c} \\ Q_a^{(n)} & = \dfrac{\tilde{Q}_a^{(n)}}{\left\langle \tilde{Q}_a^{(n)} \right\rangle_a} \end{cases} \tag{1}$$

where, in the left-hand bracket, the calculation of the fitness and complexity parameters for all countries and activities is shown, while in the right-hand one is the following normalisation step. The non-linear structure of the algorithm causes the activities in the baskets of less competitive countries (i.e. with low fitness) to be assigned a low level of complexity. The most competitive countries turn out to be those with more diversified activity baskets. Given the convergence properties of the algorithm, discussed in Pugliese et al. [17], we do not consider the complexity values but their rankings. In particular, the ranking are computed using the most recent 5-year aggregate matrices given the years of the data we considered in the analysis: thus, we use $\mathbf{M}_{c\tau}(5, 2017)$ for green patents and $\mathbf{M}_{c\pi}(5, 2017)$ for exported products.

4 Robustness test

In the manuscript we build the green technology-product bipartite network starting with two important preliminary steps: firstly, we summed the yearly data collections at our disposal over 5 years; secondly, depending on the time lag ΔT we consider, we select specific 5-year aggregate matrices. In particular, we select the two most recent exported product matrices available to us that do not overlap each other — i.e. $\mathbf{V}(\delta, t) = \{\mathbf{V}(5, 2012); \mathbf{V}(5, 2017)\}$, where δ corresponds to the interval of years over which the individual yearly matrices are summed up (in this case 5), while the year t explicitly indicated corresponds to the last year of the interval. Since the data collections of exported products are fixed for both time lags, we select the aggregated 5-year green patent collections depending on which of the latter we consider : therefore, we select the matrices $\mathbf{W}(\delta, t) = \{\mathbf{W}(5, 2012); \mathbf{W}(5, 2017)\}$ for $\Delta T = 0$ and $\mathbf{W}(\delta, t) = \{\mathbf{W}(5, 2002); \mathbf{W}(5, 2007)\}$ for $\Delta T = 10$.
In this section we want to show that our results do not depend on the choices of the years considered nor on the parameter δ. To this aim, we conduct a robustness test in which we repeat our analysis for both different values of δ and years considered. In particular, we replicate our results for a 2-digit level of product aggregation and for the time lag $\Delta T = 0$. Considering the 10 years covered by the two 5-years summed data collections we consider in the analysis for $\Delta T = 0$ — i.e. from 2008 to 2017 — we create a dataset composed by 32 matrices (16 for green patents and 16 for exported products) aggregated at 3,4 and 10 years, so that $\delta = \{3, 5, 10\}$. The dataset is reported In Table S3: each $\mathbf{M}(\delta, t)$ in the table stands for a corresponding couple of technology-product matrices $\mathbf{W}(\delta, t) - \mathbf{V}(\delta, t)$ for which we process the full analysis, meaning RCA, assist matrix and null model computations. We consider as a benchmark of this test the 46 links validated at a 95% level of significance in the manuscript. The results we obtain can be summarized as follows:

- Considering only the aggregation over 3-year intervals, on average 73% of the 46 links are present at a 95% significance level. This percentage increases to 87% if we consider a 90% level of significance for the 3-year results.
- Considering only the aggregation over 4-year intervals, on average 80% of the 46 links are present at a 95% significance level. This percentage increases to 92% if we consider a 90% level of significance for the 4-year results.
- 85% of the 46 links are present at a 95% significance level for the unique pair of technology-product matrices with the 10-year time aggregation. This percentage increases to 98% (45 links out of 46) if we consider a 90% level of significance for the 10-year result.

Based on the above summary, we consider the robustness test successful. Therefore, we interpret the results reported in the manuscript as showing a real link of interdependence between the acquisition of green technological capabilities and the development of productive ones.

**Time aggregation δ**	**Data collections $\mathbf{M}(\delta, \mathbf{t})$**
3	M(3, 2010), M(3, 2011), M(3, 2012), M(3, 2013)<br>M(3, 2014), M(3, 2015), M(3, 2016), M(3, 2017)
4	M(4, 2011), M(4, 2012), M(4, 2013), M(4, 2014)<br>M(4, 2015), M(4, 2016), M(4, 2017)
10	M(10,2017)

Table S3: Composition of the dataset we use for the robustness test of our results. Since we consider the time lag $\Delta T = 0$, data collections refer to both green patents and exported products.

References

[1] Antoine Dechezleprêtre, Matthieu Glachant, Ivan Haščič, Nick Johnstone, and Yann Ménière. Invention and transfer of climate change–mitigation technologies: a global analysis. *Review of Environmental Economics and Policy*, 5(1):109–130, 2011.

[2] Ivan Haščič and Mauro Migotto. Measuring environmental innovation using patent data. oecd. Technical report, OECD, 2015.

[3] N. Barbieri, F. Perruchas, and D. Consoli. Specialization, diversification, and environmental technology life cycle. *Economic Geography*, 96(2):161–186, mar 2020. doi: 10.1080/00130095.2020.1721279.

[4] A. Sbardella, F. Perruchas, L. Napolitano, N. Barbieri, and D. Consoli. Green technology fitness. *Entropy*, 20(10):776, 2018.

[5] N. Barbieri, A. Marzucchi, and U. Rizzo. Knowledge sources and impacts on subsequent inventions: Do green technologies differ from non-green ones? *Research Policy*, 49(2):103901, 2020.

[6] European Patenting Office. Epo–finding sustainable technologies in patents, 2013.

[7] USPTO. CPC section Y. `https://www.uspto.gov/web/patents/classification/cpc/html/cpc-Y.html`.

[8] S. Maraut, H. Dernis, C. Webb, V. Spiezia, and D. Guellec. The OECD REGPAT database, 2008.

[9] United Nations Environmental Program (UNEP). UNEP, Climate Change Mitigation definition, 2016.

[10] L. Napolitano, A. Sbardella, D. Consoli, N. Barbieri, and F. Perruchas. Green innovation and income inequality: A complex system analysis. *Structural Change and Economic Dynamics*, 2022.

[11] N. Barbieri, D. Consoli, L. Napolitano, F. Perruchas, E. Pugliese, and A. Sbardella. Regional technological capabilities and green opportunities in europe. *LEM Papers Series*, 2021.

[12] UN Comtrade. UN Comtrade database. `https://comtrade.un.org/`.

[13] D. Mazzilli, A. Tacchella, and L. Pietronero. In preparation. *None*, 2022.

[14] A. Tacchella, D. Mazzilli, and L. Pietronero. A dynamical systems approach to gross domestic product forecasting. *Nature Physics*, 14(8):861–865, 2018.

[15] A. Tacchella, M. Cristelli, G. Caldarelli, A. Gabrielli, and L. Pietronero. A new metrics for countries' fitness and products' complexity. *Scientific Reports*, 723:1–7, 2012.

[16] A. Tacchella, M. Cristelli, G. Caldarelli, A. Gabrielli, and L. Pietronero. Economic complexity: Conceptual grounding of a new metrics for global competitiveness. *Journal of Economic Dynamics and Control*, 37(8): 1683–1691, 2013. doi: 10.1016/j.jedc.2013.04.006.

[17] E. Pugliese, A. Zaccaria, and L. Pietronero. On the convergence of the fitness-complexity algorithm. *The European Physical Journal Special Topics*, 225(10):1893–1911, 2016.
